# Phytochemical investigation of *Aloe pulcherrima* roots and evaluation for its antibacterial and antiplasmodial activities

**DOI:** 10.1371/journal.pone.0173882

**Published:** 2017-03-23

**Authors:** Dele Abdissa, Girma Geleta, Ketema Bacha, Negera Abdissa

**Affiliations:** 1 Department of Chemistry, College of Natural Sciences, Jimma University, Jimma, Ethiopia; 2 Department of Biology, College of Natural Sciences, Jimma University, Jimma, Ethiopia; University of British Columbia, CANADA

## Abstract

Medicinal plants with documented traditional uses remain an important source for the treatment of a wide range of ailments. Evidence shows that majority of the Ethiopian population are still dependent on traditional medicine. *Aloe pulcherrima* Gilbert & Sebsebe is one of the endemic *Aloe* species traditionally used for the treatment of malaria and wound healing in central, Southern and Northern part of Ethiopia. The aim of the current study was, therefore, to isolate active compounds from roots of *A*. *pulcherrima* and evaluate for their antibacterial and antiplasmodial activities using standard test strains. Bioassay-guided sequential extraction and column chrom-atographic separation were employed for the isolation of bioactive pure compounds. The structures of the compounds were determined by 1D and 2D NMR spectro-scopic techniques. Disk diffusion method was employed to evaluate the antibacterial activities of the isolated compounds against four bacterial strains specifically (*Staphylococcus aureus* ATCC 25923, *Bacillus subtilis* ATCC 6633, *Escherichia coli* ATCC 35218, *Pseudomonas aeruginosa* ATCC 27853). The malaria SYBR Green I-based *in vitro* assay technique was used for *in vitro* antiplasmodial activity evaluation of the compounds against chloroquine resistant (D6) and -sensitive (W2) strains of *P*. *falciparum*. Three compounds, chrysophanol, aloesaponarin I and aloesaponarin II were isolated from the acetone extracts of roots of *A*. *pulcherrima*. Evaluation of antibacterial activities revealed that aloesaponarin I and aloesaponarin II had significant activities against all the bacterial strains with inhibition zone diameters ranging from 18–27 mm as compared to the reference drug (gentamicin), which displayed inhibition zone diameter ranging between 20 mm (*B*. *subtilis*) and 25 mm (*P*. *aeruginosa*). The isolated compounds showed moderate *in vitro* antiplasmodial activity against both chloroquine resistant (W2) -sensitive (D6) strains. Isolation of chrysophanol, aloesaponarin I and aloesaponarin II from roots of *A*. *pulcherrima* is the first report of its kind. The finding could be used for further comprehensive evaluation of the isolated compounds for their antibacterial and antimalarial activities besides consideration of the same for potent drug development.

## Introduction

Medicinal plants represent the oldest and remain an indispensable source of novel and effective pharmaceuticals. The advent of phytochemistry and pharmaceutical chemistry has enhanced the ability to utilize active compounds isolated from plants, or their synthetic equivalents in medicine. This is mainly because of the broader degree of chemical diversity and novelty possessed by medicinal plants than any other sources [1, 2].

The development of multi-drug resistance in the microbial world is posing serious challenges in the treatment of infectious diseases caused by several arrays of pathogenic microorganisms especially in the intensive care units [3–5]. In such era of high pressure from emergence of microbial drug resistance and limited therapeutic efficacy of many of the available drugs, search for novel antibacterial and antimalarial drugs of new modes of action should be given emphasis. Because of long history of their safe use and good source of therapeutic agents applicable in traditional medication, searching for alternative potent antimicrobial substances from natural products is justifiable alternative approach for the control of many infectious diseases [3–4, 6]. This has to be supported by wise use of the available commercial drugs in order to minimize the development of new drug resistant strains associated with drug misuse.

Africa has an immensely rich biodiversity and knowledge in the use of plants to treat various ailments. WHO estimated that majority of the population in sub-Saharan Africa depend solely on traditional medicinal plants for their primary healthcare needs because of their accessibility, cheapness and socio-cultural background [7]. Traditional medicines, mainly of plant origin, are the cornerstone of healthcare systems for about 80% of the population residing in Ethiopia [8]. Despite the wide use and significant importance of medicinal plants in human and animal medication, the work done so far to evaluate the efficacy of Ethiopian traditional medicinal plants is not extensive [9, 10]. The current study is part of the ongoing effort, with special emphasis on the assessment of root extract of *Aloe pulcherrima G*ilbert & Sebsebe against selected etiological agents of bacterial and malarial diseases. The genus *Aloe* (family Asphodelaceae) is the largest genus in the family comprising over 500 species [11]. *Aloe* in Ethiopia is represented by 38 species, of which 15 are endemic [12, 13]. *A*. *pulcherrima* is one of the endemic *Aloe* species, commonly used by traditional healers for the treatment of various infectious diseases [14]. It has been widely practiced in Eastern and Southern parts of the country for wound healing and as insects (tick and mosquitoes) repellents [14, 15]. Despite its wider traditional uses, however, phytochemical analysis and evaluation for biological activities pertaining to its medicinal values are limited to its leaf latex [15]. This study was initiated to isolate, characterize and evaluate biologically active compounds from roots of *A*. *pulcherrima* and; now we are reporting the isolation of three compounds along with their antibacterial and antiplasmodial activities.

## Materials and methods

### Analytical methods and culture conditions

Analytical grade solvents (*n*-hexane, chloroform, ethyl acetate, and methanol) were used for gradient extraction and column elution. Column chromatography was performed on oxalic acid impregnated silica gel [the silica gel was deactivated by mixing 1 kg of silica gel (70–230 mesh) with 3% oxalic acid (30 g in 1 L water) and allowed to stand for 30 min, filtered and dried in an oven at 100°C for 45 min]. Analytical TLC was performed on pre-coated silica gel 60 F_254_ plates, and UV-254/365 nm chamber (UV-Tec) was used for detection of spots on TLC. UV spectra were recorded on a Specord S600 (Analytik Jena AG, Germany). ^1^H NMR (600 MHz) and ^13^C NMR (150 MHz) were recorded on a Bruker Avance 500 spectrometer using the residual solvent peaks as a reference. HSQC and HMBC spectra were obtained using the standard Bruker software. Mueller Hinton agar and nutrient broth were used for antibacterial activity test while Indochina W2 (chloroquine-resistant) and the Sierra Leone D6 (chloroquine-sensitive) *Plasmodium falciparum* strains were used for antiplasmodial activity test.

### Collection and preparation of plant material

Root of *A*. *pulcherrima*, whose vernacular name is ‘*Hargisa dhala*‘ (Afaan Oromo), was collected from uncultivated land of ‘Saka Chokorsa’ district of Jimma zone (located at 7°40′N 36°50′E latitude and longitude, respectively), South Western Ethiopia, in April, 2015. This research undertaking is part of an ongoing study being carried out on plants of Asphodelacae family found in Ethiopia. *A*. *pulcherrima* is a wild plant collected from none protected area of the study site. Furthermore, this study did not involve endangered or protected species. Accordingly, no specific permission was required for collection and investigation of the aforementioned plants. Identification of the plant was made by professional botanist at Jimma University (Dr. Kitessa Hundera), and a voucher specimen was deposited at the Herbarium of Biology Department, Jimma University under voucher number AP001/2015. Roots of the plant were air-dried and fine powdered to ease the subsequent extraction processes.

### Extraction and isolation

The air-dried root samples of *A*. *pulcherrima* (810 g) were sequentially extracted with *n*-hexane, chloroform, acetone and methanol (2 x 2 L each) by percolation at room temperature. The extracts were concentrated using rota vapour under reduced pressure to yield a brown crude products weighing 2.0 g (0.25%), 3.2 g (0.39%), 4.9 (0.61%) and 4 g (0.49%), respectively. The extracts were screened for their antibacterial activity against four bacteria strains namely *S*. *aureus* (ATCC 25923), *E*. *coli* (ATCC 35218), *P*. *aeruginosa* (ATCC 27853) and *B*. *subtilis* (ATCC 6633), and extract with better activity was subjected to column chromatography. Based on the antibacterial activity of the acetone extract, it was further subjected to column chromatography (column size: 60 cm length and 4 cm diameter) on oxalic acid impregnated silica gel (200 g) eluting with *n*-hexane containing increasing amounts of ethyl acetate to afford 24 major fractions (*ca*. 200 mL each). Fractions 8–10 (2% ethyl acetate in *n*-hexane) that showed yellow fluorescing spots were combined and further purified using column chromatography (column size: 40 cm length and 2 cm diameter), eluted with increasing gradient of ethyl acetate in *n*-hexane to give chrysophanol (**1**, 8.3 mg). Fractions 20–29 (10–20% ethyl acetate in *n*-hexane) contained mixtures and showed two major fluorescing compounds when viewed under UV light at 366 nm, which were combined and subsequently subjected to repetitive column chromatography (column size: 60 cm length and 2 cm diameter), eluted with increasing gradient of ethyl acetate in *n*-hexane to yield aloesaponarin II (**3**, 14.4 mg) and aloesaponarin I (**2**, 16.3 mg) (S1 Fig).

### Evaluation for antibacterial activity

The *in vitro* antibacterial activity of the crude extracts and isolated compounds were determined against four bacteria strains listed above. The antibacterial activities of the crude extracts and pure compounds against these bacteria strains were carried out following a standard procedure as used earlier by Dagne *et al* [16]. The strains were first activated on 5% sheep red blood agar plates at 37°C for 24 hr prior to inoculation onto the nutrient agar. Some colonies of the cultured bacteria were transferred into a nutrient broth and incubated until adequate growth obtained. The cell density was adjusted to turbidity equivalence of 0.5 on McFarland scale through dilution of the activated culture using sterile nutrient broth until optical density of the diluted culture falls between 0.080–0.100 at 625 nm. The bacterial culture was then streaked onto Muller Hinton agar plate with a sterile cotton swab to obtain a uniform thick lawn of growth. Sterile paper discs (6mm diameter, Whatman No.3) were separately soaked in a pre-prepared crude extracts (whose stock solutions were prepared at concentration of 200 mg/mL by dissolving 0.2 g of the crude extracts separately in DMSO) and pure compounds (at concentration of 50 mg/mL in DMSO) before aseptically placed on an already inoculated Muller Hinton agar plate. It was allowed to diffuse for five minutes at ambient temperature and then incubated at 37^°^C for 24 hr. Gentamicin (10 μg) and DMSO were used as positive and negative controls, respectively. Finally, the antibacterial activity was evaluated by measuring diameter of zone of growth inhibitions (mm) using transparent ruler after 24 hr of incubation.

### Evaluation for antiplasmodial activity

Two strains of *P*. *falciparum*, the Indochina W2 (chloroquine-resistant) and the Sierra Leone D6 (chloroquine-sensitive), donated by the Division of Experimental Therapeutics, Walter Reed Army Institute of Research, Washington DC, were maintained in continuous culture in RPMI 1640 medium supplemented with 5% washed human A+ erythrocytes, 11 mM glucose, 25 mM HEPES, 32 nM NaHCO_3_, 29 μM hypoxanthine, and 10% heat-inactivated A+ human plasma (literally called tissue culture medium) to attain replication robustness prior to assays [17]. The culture-adapted *P*. *falciparum* were added onto the plate containing dose range of drugs and incubated in a gas mixture (5% CO_2_, 5% O_2_, and 90% N_2_) at 37°C for 72 hr and frozen at –80°C. After thawing, lysis buffer [20 mM Tris-HCl pH 7.5, 5 mM EDTA, 0.008% (wt/vol) saponin, and 0.08% wt/vol Triton X-100], containing SYBR green I (1 x final concentration) was added directly to the plates and gently mixed using the Beckman Coulter Biomek 2000 automated laboratory workstation (Beckman Counter, Inc., Fullerton, CA). The plates were then incubated for 5–15 min at ambient temperature in the dark. Parasite growth inhibition was quantified by measuring the per-well relative fluorescence units (RFU) of SYBR green 1 dye using the Tecan Genios Plus (Tecan US, Inc., Durham, NC) with excitation and emission wave-lengths of 485 nm and 535 nm, respectively, and with the gain set at 60. Differential counts of relative fluorescence units (RFUs) were used in calculating IC_50_ values for each drug using Prism 4.0 windows software. A minimum of three separate determinations were carried out for each sample. The reference anti-malarial drug, chloroquine, was tested along the test compounds.

## Results and discussion

### Characterization of the isolated compounds

**Fig 1:**
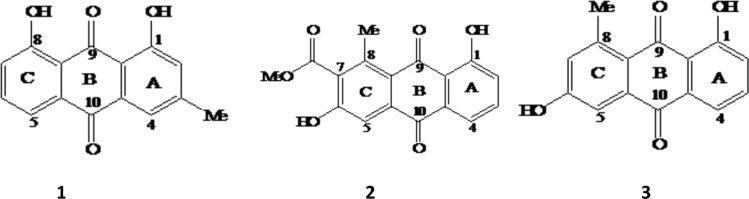
The structures of chrysophanol (1), aloesaponarin I (2) and aloesaponarin II (3) isolated from root of *A*. *pulcherrima*.

Of the four solvents used for extraction of the air dried roots of *A*. *pulcherrima*, acetone resulted in good extraction product, and the acetone extract also showed superior antibacterial activity (S1 Table, to be discussed in details in the later section). Following its activity, the acetone extract was further subjected to column chromatography on oxalic acid impregnated silica gel and has resulted in the isolation of three anthraquinones: labelled as chrysophanol (**1**), aloesaponarin I (**2**) and aloesaponarin II (**3**) (Fig 1).

Compound **1** was isolated as a yellow amorphous powder with an R_f_ value of 0.68 (8% ethyl acetate in *n*-hexane). In ^1^H NMR spectrum, two highly downfield shifted signals at δ_H_ 12.13 and 12.02 due to chelated hydroxyl protons involved in hydrogen bonding and the downfield shifted carbonyl signals at δ_C_ 194.4 and 184.6 in ^13^C NMR spectrum (Table 1) clearly indicating the presence of a 1,8-dihydroxyanthraquinone moiety [18]. The ^1^H NMR spectrum further showed five aromatic and three methyl protons. Three mutually coupled aromatic protons at δ_H_ 7.70 (*d*, *J* = 7.6 Hz), 7.80 (*t*, *J* = 7.8 Hz) and 7.37 (*d*, *J* = 8.2 Hz) with ABX spin system were assigned to H-5, H-6 and H-7 of mono-substituted ring C of a 1,8-dihydroxyanthraquinone, respectively. The presence of two *meta*-coupled aromatic protons resonating at δ_H_ 7.21 (*d*, *J* = 2.4 Hz) and 7.55 (*d*, *J* = 2.4 Hz) assigned to H-2 and H-4, respectively, with the biosynthetically expected aromatic methyl (δ_H_ 2.46) group being at C-3 of the di-substituted ring A. The ^1^H NMR data, therefore, suggested that compound **1** is 1,8-dihydroxy-3-methyl anthraquinone. In consistency with the aforementioned spectral data, the ^13^C NMR spectrum revealed 15 carbon signals; five aromatic methines (δ_C_ 123.6, 122.4, 127.3, 127.6 and 140.4), seven aromatic quaternary carbons (δ_C_ 116.9, 119.0, 136.2, 136.4, 152.2, 164.6 and 164.8), two carbonyl carbons (δ_C_ 194.4 and 184.6) and a methyl (δ_C_ 24.7) group. Based on these and comparison with the data in the literature [19], compound **1** was identified as 1,8-dihydroxy-3-methylanthracene-9,10-dione trivial name chrysophanol (**1**). Compound **1** is widely distributed in different plant families including family Asphodelaceae [20], Rhamnaceae, Rubiaceae and Polygonaceae [16, 19]. Apart from higher plants, it has also been reported from some fungi, such as *Penicillium islandicum* Sopp, as fungal metabolite [20].

**Table 1:** ^1^H (500 MHz) and ^13^C (125 MHz) NMR Spectral data for compounds 1, 2 and 3 isolated from root of *A*. *pulcherrima*.

Position	1		2		3	
	δ_H_ (*m*, *J* in Hz)	δ_C_	δ_H_ (*m*, *J* in Hz)	δ_C_	δ_H_ (*m*, *J* in Hz)	δ_C_
**1**		164.8		161.5		163.6
**1a**		116.9		116.5		1115.3
**2**	7.21 (*d*, 2.4, 1H)	127.3	7.27 (*d*, 8.3, 1H)	124.3	7.31 (*d*, 8.2, 1H)	125.2
**3**		152.2	7.67 (*t*, 7.9, 1H)	136.1	7.62 (*t*, 7.6, 1H)	136
**4**	7.55 (*d*, 2.4, 1H)	123.6	7.59 (*d*, 7.6, 1H)	118.3	7.77 (*d*, 7.6, 1H)	119.1
**4a**		136.2		132.6		124.6
**5**	7.70 (*d*, 7.6, 1H)	122.4	7.41 (*d*, 2.1, 1H)	112.1	7.80 (*s*, 1H)	115.2
**5a**		136.4		137		138.9
**6**	7.80 (*t*, 7.8, 1H)	140.4		162.4		162.6
**7**	7.37 (*d*, 8.2, 1H)	127.6	7.00 (*d*, 2.1, 1H)	124.6		132.8
**8**		164.6		145.5		148.1
**8a**		119		122.5		121.2
**9**		194.4		189.4		189.7
**10**		184.6		182.3		182.3
**CH**_**3**_	2.44 (*s*, 3H)	24.7	2.66 (*s*, 3H)	23.6	2.98 (*s*, 3H)	22
**1-OH**	12.13 (s, 1H)		12.93 (*s*, 1H)		12.93 (*s*, 1H)	
**8-OH**	12.02 (s, 1H)					
**OCH3**					4.06 (*s*, 3H)	53.3
**CO**						170.7

(Compound **1** and **2** in CDCl_3_; Compound **3** in DMSO-*d*_6_)

Compound **2** was isolated as yellow amorphous solids with an R_f_ value of 0.50 (in 20% ethyl acetate in *n*-hexane). It has UV-VIS (MeOH) λ_max_ at 220, 272, 310 and 420 nm. The ^1^H NMR (500 MHz) showed highly deshielded signal at δ_H_ 12.93 due to chelation of the hydroxyl proton involved in hydrogen bonding at C-1 (δ_C_ 163.6), a three-proton singlet at δ_H_ 4.06 (δ_C_ 53.3) and a shielded carbonyl (δ_C_ 170.7), indicating the presence of a methoxyl ester. The downfield shifted methyl signal observed at δ_H_ 2.98 (δ_C_ 22.0) due to the *peri*-effect allowed its placement at C-8 (δ_C_ 148.1). In addition, the ^1^H NMR spectrum further showed the presence of three mutually coupled aromatic protons with an ABX pattern centered at δ_H_ 7.31 (*d*, *J* = 8.2 Hz, H-2), 7.62 (*t*, *J* = 7.6 Hz, H-3) and 7.77 (*d*, *J* = 7.6 Hz, H-4) of ring A as in compound **3** (discussed in the next section). In ring C, the singlet aromatic proton at δ_H_ 7.80 showed HMBC correlation with C-6, C-7, C-8a and C-10 which is in agreement with its placement at C-5 of tri-substituted (C-6, C-7, C-8) in ring C. The substituents in this ring, a methyl group (δ_H_ 2.98 at C-8), a hydroxyl group (at C-6) and carbomethoxy group (at C-7) are evident from the NMR spectra (Table 1).

Furthermore, the ^13^C NMR spectral data (Table 1) revealed the presence of seventeen carbon atom; three carbonyls (δ_C_ 189.7, 182.3 and 170.7), four aromatic methine carbons (δ_C_ 136.0, 125.2, 119.1 and 115.2), two oxygenated aromatic quaternary carbons (δ_C_ 163.6 and 162.6), six non-oxygenated aromatic quaternary carbons (δ_C_ 148.1, 138.9, 132.8, 124.6, 121.2 and 115.3), a methoxyl (δ_C_ 53.3) and a methyl (δ_C_ 22.0) carbons. Based on the above spectral data, compound **2** was identified as 3,8-dihydroxy-1-methyl-2-anthraquinoic acid methyl ester, trivial name aloesaponarin I (**2**). It was previously reported from the stem of *A*. *saponaria* [21].

**Compound 3** was a yellow crystalline solid characterized by R_f_ value of 0.37 (in 50% ethyl acetate in *n*-hexane) and melting point of 190–192 ^0^C. It has UV-VIS (MeOH) λ_max_ at 226, 272, 285 and 427 nm, an indication for the presence of anthraquinone moiety. The presence of 15 carbon signals in ^13^C NMR spectrum and five aromatic proton signals in ^1^H NMR (Table 1) suggested that it is isomeric to compound **1**. The ^1^H NMR spectrum revealed similar spectral features to that of compound **1**; three mutually coupled aromatic protons of ABX spin pattern centered at δ_H_ 7.27 (*d*, *J* = 8.3 Hz, 1H), 7.67 (*t*, *J* = 7.9 Hz, 1H) and 7.59 (*d*, *J* = 7.6 Hz, 1H) and two *meta*-coupled protons resonating at δ_H_ 7.41 (*d*, *J* = 2.1 Hz, 1H) and 7.00 (*d*, *J* = 2.1 Hz, 1H). The ^13^C NMR spectrum showed 15 carbon signals; methines, seven aromatic quaternary carbons, two carbonyl carbons and a methyl group. The only notable difference is that of a methyl group down-field shifted (δ_H_ 2.66) in compound **3** indicating its *peri*-position to carbonyl group. In addition, the presence of only one chelated hydroxyl group (at δ_H_ 12.93), allowed the unequivocal placement of the methyl group at C-8. This was further supported by the HMBC ^4^*J*_C,H_ correlation observed between methyl protons and carbonyl carbon, C-9 (δ_C_ 189.4). Therefore, the structure of compound **3** was characterized as 1,6-dihydroxy-8-methylanthracene-9, 10-dione, trivial name aloesaponarin II (**3**). This compound has been reported from several other *Aloe* species, including stem of *A*. *saponaria* Haw. [22] and roots of other *Aloe* species [23] but not from root of *A*. *pulcherrima*, which makes our finding the first report of its kind. Besides *Aloe* species, aloesaponarin II (**3**) was reported from the culture broth of marine actinomycetes [22] along other anthraquinone (6-dihydroxy-8-hydroxymethyl-anthraquinone). According to Cui and co-workers [24], aloesaponarin II (**3**) was first reported from *A*. *saponaria* Haw. I. It was later isolated from a recombinant and terrestrial *Streptomycete*, suggesting the wide distribution of the same compound both in plants and microbes. This ensures its accessibility for sustainable utilization of the same compound for treatment of various ailments.

### Antibacterial and antiplasmodial activities of the compounds

Quinones, including anthraquinones, have gained great toxicological and pharmaco-logical interest due to their chemical structure (the quinone nucleus), allowing them to involve in multiple biological oxidative processes [25]. Accordingly, the isolated compounds were evaluated for their *in vitro* antibacterial and antiplasmodial activities and the results were promising (Table 2). The antibacterial activity tests indicated that aloesaponarin I and II (compunds **2** and **3**), compounds with closer structural feature in substituent pattern, displayed strong activity against all the test bacterial strains (inhibition zone diameter ranging between 18–27 mm). The growth inhibitory potential of compound **2** was even greater than the reference drug (gentamicin, 10 μg) against *B*. *subtilis* (Table 2). This indicates that the presence of a methoxy acetyl group at C-7 (Fig 1) may have a positive synergetic effect with a methyl group at C-8 in inhibiting the growth of bacteria. In general, the degree of growth inhibitions displayed both in Gram negative and -positive bacteria were promising although efficacies of the test compounds differ among the bacterial groups. The current observation is scientifically in support of the traditional use of medicinal plants by local community for the treatment of different bacterial ailments. Likewise, earlier reports also indicated that the leaf latex of some *A*. *pulcherrima* displayed strong antibacterial activities against both Gram positive and -negative bacterial species [15]. In the current study, in addition to antibacterial activities, the same compounds showed moderate *in vitro* antiplasmodial activity against both chloroquine-resistant (W2) and chloroquine-sensitive (D6) strains of *P*. *falciparum*. The highest activity was exhibited by compound **3** followed by compound **2** (Table 2). In general, the antiplasmodial activities of the two aloesaponarins were strong and promising againist the chloroquine-sensitive strain although all the three compunds (chrysophanol, aloesaponarin I and aloesaponarin II) had no significant activity against chloroquine resistant strain of *P*. *falciparum*.

**Table 2:** *In vitro* antibacterial and antiplasmodial activities of compounds isolated from *A*. *pulcherrima*.

Materials	Diameter of Zone of Growth Inhibition (mm)				Half maximal Inhibitory Concentration (IC_50,_ μg/mL)	
	Bacterial strain				Strains of *P*. *falciparum*	
	*B*. *subtilis*	*S*. *aureus*	*E*. *coli*	*P*. *aeruginosa*	Chloroquine-sensitive (D6)	Chloroquine- resistant (W2)
**Crude extract**	15	12	16	24	ND	ND
**1**	10	10	NI	12	21.05 ± 0.64	36.09 ± 3.32
**2**	27	18	22	21	7.80 ± 1.11	20.13 ± 5.12
**3**	22	18	21	21	5.00 ± 0.36	18.60 ± 7.10
**Gentamicin**	25	22	23	22	NA	NA
**DMSO**	NI	NI	NI	NI	NA	NA
**Chloroquine**	ND	ND	ND	ND	0.01 ± 0.001	0.22 ± 0.03

NI, No inhibition; DMSO, Dimethyl sulfoxide; NA, Not applicable; ND, Not determined

Earlier study on the antibacterial and antiplasmodial activities of aloesaponarin I (**2**) and aloesaponarin II (**3**) isolated from another *Aloe* species (*A*. *secundiflora)* [24] also indicated that these two compounds had significant antimicrobial activities, with aloesaponarin II (**3**) having strong activity against *S*. *aureus* and aloesaponarin I (**2**) with the most activity against the chloroquine-resistant (W2) strain of *P*. *falciparum*. In contrary to the same report [24], our result showed significant activity (inhibition zone diameter ≥ 21 mm) of the above two compounds against Gram negative bacteria, including *E*. *coli* and *P*. *aeruginosa*, an activity almost equal to the activity of commercial antibiotic (gentamicin). The two aloesaponarins (**2, 3**) had comparable antibacterial activities with extracts of other traditional medicinal plants reported earlier from the same study region [3, 26, 27]. However, the observed antibacterial activities were much better than acetone and ethanol extracts of *Aloe vera* leaf gel against *S*. *aureus and E*. *coli* reported elsewhere [28]. Aloesaponarin II has been used for folk medicine for the treatment of maladies in South Africa [22].

In general, the two pure compounds (aloesaponarin I and II) displayed higher activity against many of the bacterial strains tested. In some cases, however, the crude extracts appear to have even better activity as revealed by the strongest activity (inhibition zone diameter of 24 mm) against *P*. *aeruginosa* (Table 2). The better activity of the crude extract could be accounted to the synergistic interactions of several secondary metabolites (SM) present in the extract, which cannot be detected when single compounds are evaluated alone [6]. Although not specifically addressed in the current study, the mechanisms of action of secondary metabolites (SM) extracted from medicinal plants are either multi-targeted or specific to certain cellular structures. Accordingly, the narrowness or broadness of the activity depends on the target(s) of the compounds (including proteins synthesis, bio-membranes or nucleic acids). Compounds in our extract appear to have broader spectrum of bioactivities with significant activity against both Gram positive and -negative bacteria besides strong activity against *Plasmodium* strains. Such multi-target activities of many SM can explain the medical application of complex extracts from medicinal plants for more health disorders which involve several targets [6]. In general, the current study revealed potential application of extracts from roots of *A*. *pulcherrima* for the treatment of diseases due to bacteria or *Plasmodium* species having evaluated for toxicity of the pure compounds,

## Conclusion

Phytochemical investigation of the roots of *A*. *pulcherrima* has resulted in identification of three anthraquinones namely chrysophanol, aloesaponarin I (**2**) and aloesaponarin II (**3**). The isolated compounds displayed strong *in vitro* antibacterial activity against four bacteria strains (*B*. *subtilis*, *E*. *coli*, *S*. *aureus* and *P*. *aeruginosa*) with aloesaponarin I (**2**) being the most active compound against the tested bacteria strains. It even showed better activity than the reference drug (gentamicin) against *B*. *subtilis*. Although not as strong as antibacterial activity, the isolated compounds displayed moderate antiplasmodial activity against both chloroquine resistant and -sensitive malaria parasites (*P*. *falciparum*). The observed antimicrobial activities of the isolated compounds could give insight about the potentials of these anthraquinones as lead compound in development of antibacterial drugs. Further *in vivo* antibacterial and antiplasmodial tests are recommended to make conclusive decision on their potential candidacy in the development of antibacterial and antiplasmodial drugs.

## Supporting information

S1 Fig Bioassay guided isoaltion of antimicrobial compoiunds from root of *A*. *pulcherrima*. (DOC)

S1 Table Antibacterial activities of crude extracts of roots of *A*. *pulcherrima* (Conc. 200 mg/mL). (DOC)
