# Supplementary figures and images for: Phytochemical investigation of Aloe pulcherrima roots and evaluation for its antibacterial and antiplasmodial activities
Source: PLoS One. 2017 Mar 23;12(3):e0173882. doi: 10.1371/journal.pone.0173882 (PMC5363824; doi:10.1371/journal.pone.0173882)

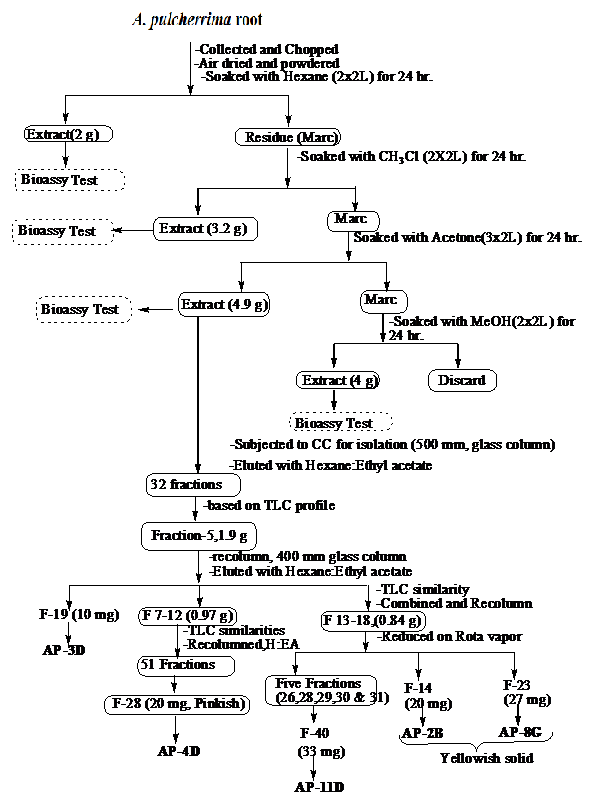


**S1Fig Bioassay guided isoaltion of antimicrobial compoiunds from root of *A. pulcherrima***

Supplement: S1 Fig — (DOC) [file pone.0173882.s001.doc]
