# Supplementary material for: Phytochemical investigation of Aloe pulcherrima roots and evaluation for its antibacterial and antiplasmodial activities
Source: PLoS One. 2017 Mar 23;12(3):e0173882. doi: 10.1371/journal.pone.0173882 (PMC5363824; doi:10.1371/journal.pone.0173882)
Supplement: S1 Table — (DOC) [file pone.0173882.s002.doc]

**S1 Table Antibacterial activities of crude extracts of roots of *A. pulcherrima* (Conc. 200 mg/mL)**

| Bacterial strain | Diameter of zone of Growth Inhibition (mm) | | | | | |
| --- | --- | --- | --- | --- | --- | --- |
|  | Hexane extract | ChloroformExtract | Acetone extract | Methanol extract | G | DMSO |
| *B. subtilis* ATCC 6633 | 9 | 20 | 15 | 9 | 20 | NA |
| *E. coli* ATCC 35218 | 10 | 9 | 16 | 8 | 20 | NA |
| *S. aureus* ATCC 25923 | 10 | 8 | 12 | 9 | 23 | NA |
| *P. aeruginosa* ATCC 27853. | 13 | 15 | 24 | 11 | 25 | NA |

Where: NA, No activity; G, Gentamicin; DMSO, Dimethyl sulfoxide
